# Nasal septal teratoma in a child

**DOI:** 10.1186/1477-7819-5-58

**Published:** 2007-05-31

**Authors:** Titus S Ibekwe, Daniel D Kokong, Bethrand A Ngwu, Oluwole A Akinyemi, Onyekwere G Nwaorgu, Effiong E Akang

**Affiliations:** 1 Department of Otorhinolaryngology, University College Hospital Ibadan, Nigeria; 2 Department of Pathology, University College Hospital Ibadan, Nigeria; 3 Department of Anesthesia, University College Hospital Ibadan, Nigeria

## Abstract

**Background:**

Teratoma is a rare developmental neoplasm that arises from totipotential tumor stem cells. Head and neck teratomas constitute about 10% of all cases. Only two cases of mature teratoma of the nasal septum have previously been documented in the world literature.

**Case presentation:**

We present a case of histologically confirmed mature teratoma arising from the nasal septum in an eighteen month old Nigerian female who presented with a history of noisy breathing associated with recurrent rhinorrhea since birth. Physical examination revealed obstruction of the right nasal cavity by a pale fleshy mass. She underwent a total surgical excision and to date, after thirty one months follow-up, she is free from recurrence.

**Conclusion:**

The prognosis for benign teratoma of the nasal septum is good following total surgical excision.

## Background

Teratoma is a developmental and sometimes congenital neoplasm that displays gross and microscopic differentiation into a wide variety of tissues representative of all three germ layers – ectoderm, mesoderm and endoderm. The presence of tissues within the lesion that are foreign to the affected sites is a distinctive feature of this rare tumor. The incidence of teratoma worldwide is about 1 in 4,000 live births [1]. It can occur in any region of the body; however teratoma involving the head and neck region is very rare and constitutes about 10% of all teratomas [2,3]. A MEDLINE search revealed only two previous cases of nasal septal teratoma [4,5]. It is the purpose of this paper to document the first case of nasal septal teratoma in Africa and the third in the World literature.

## Case presentation

An eighteen month old Nigerian female presented to our health facility with a history of noisy breathing since birth, associated with intermittent mucoid rhinorrhea, especially from the right nasal cavity. Protrusion of a pale mass in the right nasal cavity was observed by the parents prior to presentation. There was no epistaxis, fever or difficulty with feeding. The child was the product of a term pregnancy and delivery was said to be uneventful. The developmental milestones were within normal expectations.

Examination revealed a well-fed young girl with noisy respiration, afebrile and not pale. The external nasal pyramid appeared splayed; the right nasal cavity was completely obliterated by a lobulated pale mass which was firm, sensitive to touch, non-hemorrhagic and attached to the nasal septum. It was partly covered by mucoid secretions. Test aspiration of the mass was dry. The left nasal cavity, ears, throat and neck appeared normal. A nasogastric tube was easily passed through the left nasal cavity. No lesion was seen in any other parts of the body.

X-ray (occipito-frontal and post nasal space views) revealed radio-opacity of the right nasal cavity, extending posteriorly into the nasopharynx. There was no evidence of calcification of the soft tissue shadow or bony erosion of the nasal bony framework. The plain radiographs of the soft tissue neck and the chest were within normal limits. Complete blood and platelet counts were within normal range.

**Figure 1:**
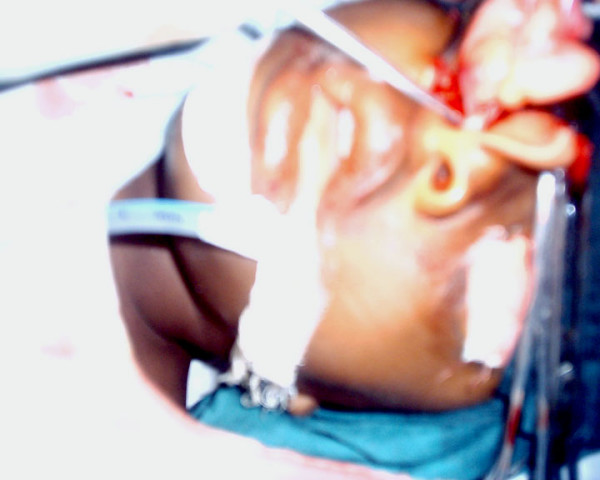
Illustrates access via a right lateral rhinotomy incision with the right ala elevated to demonstrate the right nasal teratoma completely filling the nasal cavity and attached to the nasal septum on a stalk.

Examination under general anesthesia (via oro-tracheal intubation) revealed that the right nasal mass could neither be delivered via the anterior nares nor the post nasal space, prompting a right lateral rhinotomy for adequate exposure of the mass (Figure 1). The stalk which was attached at the mid portion of the cartilaginous septum was rocked and completely excised and the rest of the mass delivered. Hemostasis was achieved and wound closed in two layers.

**Figure 2:**
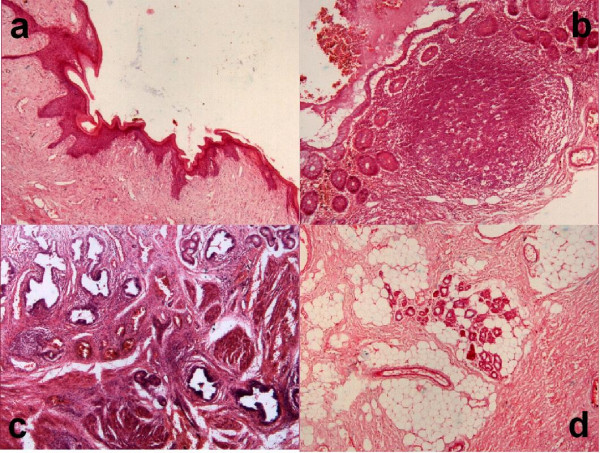
**a **– Shows a cyst lined by epidermis (hematoxylin-eosin, × 40); **b **– Shows a cyst lined by gastrointestinal epithelium with prominent lymphoid follicles (hematoxylin-eosin, × 40); **c **– Shows numerous glands lined by columnar epithelium, interspersed by fibromuscular tissue (hematoxylin-eosin, × 40); **d **– Shows mature adipose tissue with numerous sweat glands (hematoxylin-eosin, × 40)

The excised mass appeared irregular, but smooth-surfaced, measuring 6 × 5 × 3 cm and weighing 27 g. The cut surface showed a lobulated tan colored appearance. Histological examination revealed haphazardly arranged heterogeneous tissues, including cysts lined by epidermis with associated hair follicles and sweat glands, gastrointestinal epithelium, respiratory epithelium and columnar epithelium reminiscent of mammary glands. Lobules of mature adipose tissue and fibrocollagenous connective tissue were also present (Figure 2). These histological features were consistent with a mature teratoma.

The postoperative course was uneventful and the patient was discharged on the seventh day post surgery. Follow-up for the past 31 months has been symptom free.

## Discussion

'Teratoma' literally means monstrous tumor. According to Hamilton [6], the Greek word 'teraton' was first used by Virchow in the first edition of his book on tumors published in 1863. These tumors are derived from totipotential stem cells and are typically paraxial or mid-line in location. Reports from literature on teratoma in children have shown that sacrococcygeal teratoma (57%) is the most common followed by gonadal teratoma (29%) [6]. Other documented sites of occurrence includes the mediastinum (7%), the retroperitoneum (4%), the cranial cavity (3%) and the neck region (3%) [6,7].

Strictly speaking, head and neck teratomas are very rare and the localization in the nasal cavity is very unusual [2,3]. Only two previously reported cases of teratoma arising from the nasal septum were found in the world English literature following a MEDLINE search [4,5]. None has been reported in Africa. Most of the reported cases are of nasopharyngeal and cervical origin [5].

Generally, teratomas are more common in females than males [8]. However, it has been established that there is no sex predilection in head and neck teratomas [1]. The majority of cases are diagnosed during infancy, and some immediately after delivery. The quality of antenatal obstetric care and the mode of presentation of the tumor are important aids to early detection of head and neck teratomas. Poor antenatal and obstetric care might be responsible for the delay in making diagnosis for our patient since a thorough nasal examination following the noisy breathing observed at birth would have revealed a nasal mass prompting referral to ENT surgeons.

There are three main histological types of teratomas which include mature (benign), immature (malignant) and monodermal (highly specialized) teratomas [9]. In our case, it was a mature (benign) type, similar to the two cases reported earlier by Takehara *etal *[4] and Stretharan and Prepaqeran [5]. All the histological variants are known to elaborate/synthesize alpha-fetoprotein which gives rise to polyhydramnios in 18% of such pregnancies [8]. This information is useful in making *in utero *diagnosis of this tumor [10].

Beside ultrasonography, there are other radiological tools that are necessary to confirm or rule out other possible differential diagnoses prior to the histological diagnosis. Plain radiograph (X-ray) of the upper aero-digestive system would reveal a soft tissue shadow as seen in this case. Teratoma shows some tissue calcification in about 16% of cases [11]. The calcification depicts the presence of highly mineralized well differentiated tissues, including teeth and bones.

Computed tomographic scan would exclude bony erosion or defect at the base of the skull as it would be seen in encephalocele or in malignant tumors. The origin of antro-choanal polyps would better be defined by computed tomographic scan. Magnetic resonance imaging is useful in both *in utero *and postpartum diagnosis because of the good resolution for the soft tissues. In the developing countries, like ours, poverty is limitation to the use of these high resolution radiological investigative tools. For instance, the index case was requested to do a cranio-facial CT-scan as part of the work-up for surgery, but could not afford it.

Fine needle aspiration cytology and tissue histology would further differentiate nasal teratoma from other neoplasias, such as nasal glioma, choristoma, rhabdomyoma, and granulomatous lesions. Histological examination remains the gold standard for the confirmation of this lesion.

The principles of treatment for nasal and other upper airway teratomas include early intervention, through establishment and maintenance of airway, total surgical excision of the tumor, adjuvant therapy (chemo-radiation) with aggressive monitoring of the patient within the first 2 years and then close monitoring for life in adults if there is evidence of malignant transformation [11].

Over three decades ago, Hawkins and Park [12] reported that there is about 7% chance of mortality during a total excision of the teratoma of the upper airways. However, non excision of the tumor would ultimately lead to death because of the rapid growth rate.

Our patient had benign unilateral nasal teratoma, which was managed through surgical excision alone with a good outcome. An external approach (lateral rhinotomy) was used here, since we lack the equipments for less invasive and more sophisticated transnasal endoscopic technique. This is similar to the mode of management adopted by Takehara *et al *[4] in the management of the first reported case in the world literature.

Streetharan and Prepageran [5] managed the second reported case of benign nasal septal teratoma with total tumor excision using the endoscopic technique with good outcome. Therefore, we can conclude that the prognosis for benign teratoma is good with total excision.

## Competing interests

The author(s) declare that they have no competing interests.

## Authors' contributions

**TI **conceived the idea of reporting the case.

**DDK **– assisted during the surgery and in the literature search.

**BAN **– the pathology Senior Registrar that prepared the histology slides and contributed in literature search and manuscript preparation.

**OAA **– the consultant anesthetist that anesthetized the patient and also contributed in the manuscript preparation.

**OGN **– the principal surgeon, who also proof-read and made necessary corrections on the manuscript.

**EEA **– read the slides and processed the microfilms for the slides to illustrate the various components of the teratoma.

All authors read and approved the final manuscript.
